# Evaluation of the antimycobacterial activity of crude extracts and solvent fractions of selected Ethiopian medicinal plants

**DOI:** 10.1186/s12906-017-1563-0

**Published:** 2017-03-08

**Authors:** Wubayehu Kahaliw, Abraham Aseffa, Markos Abebe, Mekonen Teferi, Ephrem Engidawork

**Affiliations:** 1 Department of Pharmacology, School of Pharmacy, College of Medicine and Health Sciences, University of Gondar, P. O. Box: 196, Gondar, Ethiopia; 2 Armauer Hanson Research Institute, P. O. Box: 1005, Addis Ababa, Ethiopia; 3 Department of Pharmacology and Clinical Pharmacy, School of Pharmacy, College of Health Sciences, Addis Ababa University, P. O. Box: 1176, Addis Ababa, Ethiopia

**Keywords:** Tuberculosis, Resazurin, Antimycobacterial activity, Solvent fractions, Crude extract, Medicinal plants

## Abstract

**Background:**

Tuberculosis (TB) is a global health problem complicated by drug resistance and human immunodeficiency virus that has dramatically increased active TB. Several medicinal plants are used traditionally to treat TB in Ethiopia and investigating these plants is required as plants are an alternative source for development of new anti-TB drugs. The purpose of this study was to investigate antimycobacterial activity of crude extract of Carissa edulis, Otostegia integrifolia, Persea americana, Pterolobium stellatum and Vernonia amygdalina as well as fractions of the most active crude extract.

**Methods:**

The effect of various doses of the crude extracts as well as solvent fractions on *M. tuberculosis* H37Rv and/or MDR-TB clinical isolate was determined using broth microdilution and microtiter resazurin assay methods. Minimum inhibitory concentration was determined by CFU count and resazurin color change observation.

**Results:**

Chloroform and 80% methanol extracts of *P. stellatum* and *O. integrifolia* as well as 80% methanol and acetone extracts of *P. americana* had significant antimycobacterial activity (*p* < 0.001) against *M. tuberculosis* H37Rv. Chloroform extract of *V. amygdalina* and *C. edulis* didn’t, however, show any significant activity compared to negative controls. *P. stellatum* chloroform extract was the most active on *M. tuberculosis* H37Rv (MIC 0.039 mg/ml) and AOZ8W-4 (MDR-TB clinical isolate) (MIC = 0.078 mg/ml). Ethyl acetate fraction of *P. stellatum* chloroform extract was the most active fraction.

**Conclusion:**

*P. stellatum, O. integrifolia* and *P. americana* were found to be endowed with antimycobacterial activity. However, *P. stellatum* appears to be the most promising plant based on criteria set by different studies. Ethyl acetate fraction of *P. stellatum* was found to be the most active and future studies should involve this fraction.

## Background

*Mycobacterium tuberculosis,* an obligate aerobe belonging to the *M. tuberculosis* complex (also include *M. bovis, M. africanum and M. microti*), is the most important cause of TB in humans. In addition to *M. tuberculosis* complex, *Mycobacterium avium* complex (include *M. avium, M. intracellularae, and M. kansasii*) can also cause closely related mycobacterium diseases in AIDS patients and is difficult to distinguish as a group clinically [1].

In 1993, during the world TB day, WHO declared TB as a ‘global emergency,’ which requires emergency action and launched several programs to combat the disease, including the search for newer remedies and/or anti-TB agents to complement currently used agents [2]. In the year 2014, TB killed 1.5 million people (1.1 million HIV-negative and 0.4 million HIV-positive) and 9.6 million new cases of active tuberculosis globally caused by TB [3].

Mycobacteria are slow growing organisms that require administration of a combination of drugs for extended periods to achieve effective therapy and to prevent the emergence of resistance. The risk of adverse reactions therefore must be a major consideration in drug selection. Apart from significant toxicity, lengthy therapy also creates poor patient compliance. Non-compliance is a frequent cause of a deadly multidrug resistant TB (MDR-TB) and extensively drug resistant TB (XDR-TB). Drug toxicity coupled with the problem of mycobacterial persistence highlights the need to develop novel TB drugs that are active against drug resistant bacteria and kill persistent bacteria as well as shorten the length of TB treatment [4].

Natural products, either as pure compounds or as standardized plant extracts, provide unlimited opportunities for new drug leads because of the unmatched availability of chemical diversity [5]. Scientific interest in medicinal plant has grown in recent times because natural products are evolutionary shaped drugs or drug‐like molecules. Nature’s biosynthetic machinery produces innumerate natural products with distinct biological properties that make them valuable as inhibitors or promoters of biological action [4, 6]. Moreover, higher plant extracts have been considered as promising sources of novel anti-TB leads [7]. This has prompted us to investigate selected Ethiopian medicinal plants, including *Carissa edulis* Vahl (Apocynaceae), *Otostegia integrifolia* Benth (Lamiaceae (Labiatae)), *Persea Americana* Mill (Lauraceae), *Pterolobium stellatum* (Forsk.) Brenan. (Fabaceae), and *Vernonia amygdalina* Del. (Asteraceae) for possible anti-TB activity. The plants have been traditionally used to treat respiratory or lung-related diseases, including TB for a very long time [8–11]. The present study attempted to screen the antmycobacterial activity of different solvent extract of these five Ethiopian medicinal plants, and *Pterolobium stellatum* was found to be the most promising plant for further investigation.

## Methods

### Plant collection

The roots of *O. integrifolia, P. stellatum,* and *C. edulis* were collected from an area near *Angereb* River, Gondar town, North West Ethiopia, about 730 km away from the capital, Addis Ababa. The root of *V. amygdalina* and the leaves of *P. americana* were collected from Bure town, North West Ethiopia, about 400 km far from Addis Ababa. The plants were authenticated by a taxonomist (Mr. Melaku Wondafrash) and a voucher specimen of each plant material was deposited at the National Herbarium, College of Natural and Computational Sciences, Addis Ababa University for future reference with voucher numbers Wk001, WK002, WK003, WK004 and WK005 for *O. integrifolia*, *P. stellatum*, *C. edulis*, *V. amygdalina* and *P. americana*, respectively. The plants were cleaned from dirt and soil and dried under shade for two weeks. The plants were spread out and regularly turned over to avoid fermenting and rotting. The dried root parts of plants were grinded using 0.75 mm sieve size hammer type mill, while the dried leaves were pulverized using a wooden mortar and pestle. The powdered material was weighed using an analytical balance and stored at room temperature.

### Experimental animals

Swiss albino nulliparous and non-pregnant female mice weighing 31–36 g, and age 8–12 weeks were obtained from the Department of Pharmacology and Clinical Pharmacy, School of Pharmacy, Addis Ababa University. All animals were housed in an air-conditioned room and allowed to acclimatize for one week before commencement of the study. All the experiments were conducted in accordance with internationally accepted laboratory animal use, care and guideline [12] and the study was approved by the School of Pharmacy Ethics Committee (Protocol number was 042/12/pharmacy). Before and during the experiment, mice were allowed free access to standard pellets and water ad libitum.

### Bacterial strains and inoculums preparation

Bacterial cells were *M. tuberculosis* H37Rv (ATCC no 27294, Manassas, VA) and three MDR-TB clinical isolate strains. The three MDR-TB clinical isolates were AOA8W-4, AOZ8W-4 and SO38SW-4. *M. tuberculosis* and MDR-TB strains were cultured and grown on Mycobacteria 7H11 medium following procedures described elsewhere [13]. The inoculum was then prepared by diluting cultures at 1/1000 by adding 25 μl cell culture to 25 ml medium, 7H9 broth (4.7 g of Middlebrook 7H9 broth base [Difco - Becton Diskinson], 2 ml of glycerol in 900 ml water) enriched with ADC when cooled to 47 °C [13].

### Extraction of plant materials

#### Crude extract

The air-dried, powdered roots of *P. stellatum*, *O. integrifolia*, *C. edulis* and *V. amygdalina* were exhaustively extracted with chloroform using maceration technique, while *P. americana* powdered leaves were extracted with acetone. Up on using chloroform and acetone solvents, maceration was carried out using one liter of the respective solvent for 72 h, with regular shaking. The mixture was filtered with Whatman No. 42 filter paper and the filtrate was kept at + 4°c. The marc was macerated again in the same solvent two times and filtered. The filtrates were combined evaporated under reduced pressure on a rotary evaporator (Buchi Rota Vapor R-200) and dried in oven at 40°c (Gallenkamp, England).

In parallel, the air-dried and powdered roots of *P. stellatum*, *O. integrifolia* and powdered leaves of *P. americana* were Soxhlet extracted with 80% methanol (4:1, methanol: water). The obtained extracts were filtered and evaporated under reduced pressure on a rotary evaporator and lyophilized. The extracts were kept refrigerated and away from light. Stock solutions of all extracts were prepared in DMSO at a concentration of 50 mg/ml and stored at −20 °C until use.

#### Fractionation

Antimycobacterial activity evaluation of the crude extract revealed *P. stellatum* chloroform root extract to have a better activity and further fractionation was pursued using this plant. The dried extract (8 g) was suspended in distilled water (200 ml) and then successively partitioned with n-hexane and ethyl acetate in a separatory funnel. The n-hexane and ethyl acetate fractions were concentrated under reduced pressure using rotary evaporator (Buchi Rota Vapor R-200) and dried in an oven under 40 °C, while the water fraction was dried using freeze drier.

### Acute oral toxicity test

As no toxicity data was available for any part of *P. stellatum* chloroform extract, oral toxicity study was conducted using Organization for Economic Cooperation and Development (OECD) guidelines 423 [14]. Briefly, nine mice were randomly divided into three groups of three mice per cage. Before administration of single dose of the extract, the mice were fasted for 3 h and weighed. After administration of a single dose of extract, mice were fasted for 1 h. The first group was given solvent (5% DMSO in distilled water), while the second group were given 2 g/kg (dissolved in 5% DMSO) of the chloroform root extract of *P. stellatum* orally. The mice in the third group were provided with *P. stellatum* root extract 5 g/kg dissolved in 5% DMSO after following the first two groups for 14 days. The mice were observed continuously for 1 h after administration of the extract; intermittently for 4 h, over a period of 24 h, and then frequently for 24 h for 14 days. Gross behavioral changes such as loss of appetite, hair erection, lacrimation, tremors, convulsions, salivation, diarrhea, mortality and other signs of toxicity manifestation were observed.

### In vitro antimycobacterial assay

#### Colony count assay

The antimycobacterial effect of the extracts was evaluated at concentrations ranging from 0.00244 mg/ml to 2.5 mg/ml. The test concentrations of the extracts were selected based on cytotoxicity test results in previous studies [4, 10, 15, 16]. Serial two-fold dilution of extracts was made in microtiter wells. Colony forming units (CFUs) were counted from triplicate dilutions and duplicate plates were used for each concentration of test extract and controls. The average CFUs were determined from three independent experiments. The percentage inhibition of growth was determined by dividing the CFUs of the test concentration by the CFUs of the negative control (solvent).

Each extract reconstituted with DMSO (50 mg/ml) was further diluted (5 mg/ml) with Middlebrook 7H9 broth supplemented with Middlebrook ADC. Activity was then measured and MIC was determined for the extracts as well as positive and negative controls following a method described elsewhere [13, 17]. The MIC was the concentration of extract at which 100% inhibition of mycobacterial growth was observed when compared with the growth control.

#### Resazurin indicator assay

*M. tuberculosis* H37Rv and clinical isolates of MDR-TB were cultured in Middlebrook 7H9 broth at 37 °C for two weeks in order to reach logarithmic phase growth. Test inoculums were prepared using a procedure described by Patricia and colleagues [18]. After incubation at 37 °C for 7 days, 15 μl of 0.01% resazurin solution in sterile water was added to the first growth control wells and incubated for 24 h. Once the first sets of growth controls turned pink, the dye solution was added to the second set of growth controls and the test wells, incubated for 24 h at 37 °C. Blue color in the wells containing the test compounds would indicate inhibition of growth, while pink indicates lack of inhibition of growth of *M. tuberculosis* [19]. MIC value was expressed as the lowest concentration of compound that caused 100% inhibition of mycobacterium growth [18]. All assays were run in triplicate and isoniazid was used as positive and DMSO as negative control.

### Statistical analysis

Data are expressed as mean ± standard error of mean as appropriate and Statistical analysis of all results was done using the Statistica 10 and GraphPad Prism 5 software. Analysis was done using one-way ANOVA followed by Duncan post hoc test. Level of statistical significance was set at α < 0.05.

## Results

Information pertaining the preparation of the crude extracts is depicted in Table 1.

**Table 1:** Outcome of extraction in the preparation of crude extracts

Plants	Part (s) used	Solvent	Extraction method	Raw material (g)	Yield g (%)	Physical appearance
*Otostegia integrifolia*	roots	chloroform	maceration	300	2 (0.67)	whitish powder
	roots	80% methanol	soxhlet	200	1 (0.5)	deep red gumy
*Pterolobium stellatum*	roots	chloroform	maceration	500	10 (2)	brownish resin
	roots	80% methanol	soxhlet	200	1.5 (0.75)	deep red gumy
*Persea americana*	Leaves	80% methanol	soxhlet	500	20 (4)	Oily black
		Acetone	maceration	300	3 (1)	gumy black
*Carissa edulis*	roots	chloroform	maceration	300	2 (0.67)	light green
*Vernonia amygdalina*	roots	chloroform	maceration	300	1.5 (0.5)	black powder

### Acute oral toxicity test

Acute oral administration of the chloroform extract of *P. stellatum* at different doses showed no overt signs of distress for the 14-day observation period. Moreover, there were no observable symptoms of toxicity or deaths even at a dose of 5 g/kg. This indicates that the oral LD_50_ was greater than 5 g/kg. All treatment and control group mice gained weight and no significant changes in behavior was noted, suggesting that administration of the crude extract had negligible level of toxicity on growth of the animals. In addition, gross behavioral changes such as loss of appetite, hair erection, lacrimation, tremors, convulsions, salivation, diarrhea, mortality and other signs of toxicity manifestations were not observed.

### Antimycobacterial activity testing using CFU method

Good growth of *M. tuberculosis* H37Rv was evident in the microtiter plate wells containing only liquid Middlebrook 7H9 medium and in the universal Petri dishes containing solid Mycobacteria 7H11 medium, within three to four weeks. DMSO control at 2.5 to 0.0024%, equivalent to solvent concentrations in test extracts, exhibited no inhibitory effect on mycobacterial growth as evidenced by higher CFU/ml (Table 2). Among the different concentrations of isoniazid used, total inhibition of growth of mycobacteria was observed at a concentration of 0.125 μg/ml.

**Table 2:** Antimycobacterial activity of experimental plants using colony count method

Concentration of extract (mg/ml)	CFU/ml									
	DMSO ƚ	*P. stellatum* CHCl_3_ extract	*P. stellatum* MeOH extract	*O. integrifolia* CHCl_3_ extract	*O. integrifolia* MeOH extract	DMSO ǂ	*V. amygdalina* CHCl_3_ extract	*C. edulis* CHCl_3_ extract	*P. americana* MeOH extract	*P. americana* acetone extract
2.50	583	0	0	0	0	354	0	0	0	0
1.25	1194	0	0	0	0	476	0	0	72	0
0.625	2944	0	0	0	0	488	62	43	197	84
0.312	4000	0	0	0	150	742	166	168	64	282
0.156	5944	0	100	334	270	800	260	208	103	94
0.078	8444	0	277	566	650	817	242	222	66	89
0.039	9345	0	302	860	1050	871	496	435	94	344
0.019	1122	100	351	360	886	1275	1033	552	89	1028
0.009	12333	100	421	3334	1334	1517	1733	742	80	28
0.005	19861	100	450	3389	3889	958	2013	813	578	1120
0.0024	16944	100	200	3445	6389	1100	2438	1206	698	1249
Mean ± SEM	7519.45 ± 1984.82	36.36*** ± 15.21	191.00*** ± 53.90	1117.00*** ± 447.7	1329.00 *** ± 608.3	854.36 ± 351.01	767.55 ± 269.50	399.00 ± 117.99	185.55 *** ± 69.34	392.50* ± 147.7

Experiments were carried out in triplicate and results are expressed as means of three replicate experiments. ƚ = Solvent CFU for chloroform and methanol extracts of *P. stellatum* and *O. integrifolia*; ǂ = Solvent CFU for chloroform extract of *V. amygdalina* and *C. edulis* and 80% methanol and acetone extract of *P. Americana*,; MeOH = methanol. * = α < 0.05, *** = α < 0.001

Extracts showed differential effects on the proliferation of *Mycobacterium tuberculosis*. The chloroform extract of *P. stellatum* appeared to be the most active as growth was totally inhibited at concentrations as low as 0.039 mg/ml. Concentrations more than 0.3 mg/ml were required to bring about no growth with the 80% methanol extract of *P. stellatum* and *O. integrifolia* as well as with the chloroform extract of *O. integrifolia*. For the rest of the extracts, increasing concentrations (>1 mg/ml) were associated with growth arrest (Table 2).

The chloroform and 80% methanol extracts of *P. stellatum* and *O. integrifolia* as well as 80% methanol and acetone extracts of *P. americana* had significant antimycobacterial activity (α < 0.001) against *M. tuberculosis* H37Rv compared to vehicle treated group. By contrast, *V. amygdalina* and *C. edulis* chloroform extracts did not have any detectable effect. It is interesting to note that although there was complete inhibition of growth of *M. tuberculosis* H37Rv at concentrations greater than 1.25 mg/ml for *V. amygdalina* and *C. edulis* chloroform extracts, the difference failed to reach statistical significance (Table 2).

### Activity testing of crude extract on clinical isolates using Resazurin indicator method

As the chloroform extract of *P. stellatum* demonstrated the highest activity (MIC = 0.039 mg/ml) against the standard strain in the CFU method, screening of the activity of this extract against clinical isolates of MDR-TB was carried out using resazurin indicator method. The resazurin assay results are depicted in Table 3. Color readings of the growth control wells were pink, demonstrating high levels of mycobacterial growth, while wells with broth alone appeared as blue color, which demonstrated no growth and lack of contamination. The isoniazid containing control wells were pink revealing the clinical isolates were isoniazid resistant. Whilst the extract demonstrated highest activity against AOZ8W-4 strain (MIC 78 μg/ml), it had a moderate activity against both AOA8W-4 and SO38SW-4 strain (MIC 156 μg/ml).

**Table 3:** Antimycobacterial activity test of *Pterolobium stellatum* chloroform extract against MDR-TB clinical isolates

		MDR-TB clinical isolates’ suspension color		
Plant extracts and controls	Concentration (mg/ml or %)	AOA8W-4	AOZ8W-4	SO38SW-4
*P. stellatum* CHCl_3_ extract	2.500	blue	blue	blue
	1.250	blue	blue	blue
	0.625	blue	blue	blue
	0.312	blue	blue	blue
	0.156	blue	blue	blue
	0.078	pink	blue	pink
	0.039	pink	pink	pink
	0.019	pink	pink	pink
	0.009	pink	pink	pink
	0.005	pink	pink	pink
	0.0024	pink	pink	pink
DMSO control	2.5–0.0024%	Pink	Pink	Pink
Isoniazid	1 ×10^−3^	Pink	Pink	Pink
	0.5 ×10^−3^	Pink	Pink	Pink
	0.25 ×10^−3^	Pink	Pink	Pink
	0.125 ×10^−3^	Pink	Pink	Pink
	0.06 ×10^−3^	Pink	Pink	Pink
	0.03 ×10^−3^	Pink	Pink	Pink
Growth control	-	Pink	Pink	Pink
Sterility control	-	blue	blue	blue

Experiments were carried out in triplicate and results are expressed as means of three replicate experiments. - = Not applicable

### Activity testing of solvent fractions on standard strains using Resazurin method

As presented in Table 4, n-hexane and aqueous fractions were evaluated in a concentration series of 1000 to 0.977 μg/ml, while ethyl acetate fraction with 200 to 0.195 μg/ml concentrations. The microtiter wells were pink below a concentration of 15.625 μg/ml for the n-hexane fraction, indicating that this fraction was active against the standard strain (MIC = 15.625 μg/ml). By contrast, all wells were pink for the aqueous fraction, suggesting that this fraction was not active against the bacteria within the exposure concentrations used for the experiment. The ethyl acetate fraction was active against *M. tuberculosis* H37Rv (MIC = 0.195 μg/ml), indicating that this fraction is the most active fraction of *P. stellatum* chloroform extract (Table 5). As described above, all wells treated with the vehicle were pink, demonstrating no inhibitory role of the vehicle.

**Table 4:** Test suspension colors obtained from fractions tested against *Mycobacterium tuberculosis* H37Rv

n-hexane		Ethyl Acetate		Aqueous		Isoniazid		DMSO	
Conc (μg/ml)	Color	Conc (μg/ml)	Color	Conc (μg/ml)	Color	Conc (μg/ml)	Color	Conc (%)	Color
1000	blue	200	blue	1000	pink	1	blue	2.5	pink
500	blue	100	blue	500	pink	0.5	blue	1.25	pink
250	blue	50	blue	250	pink	0.25	blue	0.625	pink
125	blue	25	blue	125	pink	0.125	blue	0.312	pink
62.5	blue	12.5	blue	62.5	pink	0.06	pink	0.156	pink
31.25	blue	6.25	blue	31.25	pink	0.03	pink	0.078	pink
15.625	blue	3.125	blue	15.625	pink	–	–	0.039	pink
7.813	pink	1.563	blue	7.813	pink	–	–	0.02	pink
3.906	pink	0.781	blue	3.906	pink	–	–	0.01	pink
1.953	pink	0.391	blue	1.953	pink	–	–	0.005	pink
0.977	pink	0.195	blue	0.977	pink	–	–	0.0024	pink

Experiments were carried out in triplicate and results are expressed as means of three replicate experiments. - = Not applicable

**Table 5:** Minimum inhibitory concentrations of solvent fractions against *Mycobacterium tuberculosis* H37Rv

Solvent fraction	Minimum Inhibitory Concentration (μg/ml)
n-hexane fraction	15.625
Ethyl acetate fraction	0.195
Aqueous fraction	+++
Isoniazid	0.125
Growth control	+++
DMSO control	+++

+++ = Growth

## Discussion

Current TB therapy consists of treatment with a combination of drugs. This combination therapy causes hepatotoxicity as the major side effect as well as development of drug resistance. To avert toxicity and reduce ineffectiveness of current anti-TB drugs, medicinal plants are considered as potential anti-tuberculosis agents that can be used in combination with the standard anti-tuberculosis drugs or alone [20]. In this study, antimycobacterial activity of crude extract of *O. integrifolia*, *V. amygdalina*, *C. edulis, P. americana*, *P. stellatum* and as well as fractions of the most active crude extract was investigated.

From the aerial parts of *O. integrifolia* otostegin A, otostegin B, 15-*epi*-otostegin B, preleoheterin, leoheterin, and related compounds, including leopersin C, 15-*epi*-leopersin C, ballonigrin, vulgarol, and 8-O-acetylharpagide were isolated and reported. In addition, the essential oil and chloroform extract of air-dried leaves of *O. integrifolia* constitute monoterpenes, sesquiterpenes, diterpenes and their derivatives were identified [21]. Phytochemical screening study report indicated that saponins, glycosides and tannins, which are known to be bioactive purgative principles were present in *V. amygdalina* extract. Flavonoids are also present in the plant that possess antioxidant activity and may play a beneficial role in cancer prevention and offer some protection against diabetes and atherosclerosis [22].

The chemical compositions of *C. edulis* have extensively been reported. Roots contain an active ingredient, carissin that may prove useful in the treatment of cancer. The twigs contain quebrachytol and cardioglycosides that are useful as an anthelmintic against tapeworm [23] and the roots contain lupeol (has antiviral activity), oleuropein, carissol and β-amyrin [24]. Major chemical constituents of *P. americana* include the following: the leaf contains volatile oil, flavonoids and coumarins; the fruit contains sesquiterpenes and carbohydrates; the seed contains fixed oil consisting of vitamins A, D_3_, alpha tocopherol and cholesterol. The fruit is a significant source of protein, monounsaturated fatty acids, vitamin A, thiamin, riboflavin, niacin, vitamin B6, vitamin C, vitamin E, folate, vitamin K, pantothenic acid, magnesium, manganese, phosphorus and the amino acids tryptophan, valine, tyrosine, threonine, phenylalanine and methionine [25]. Chemical classes present in *P. stellatum* 80% root extract are terpenoids, saponins and tannins and had antibacterial activity as reported by previous study.

The result of the study revealed that three of the experimental plants had significant anti-tuberculosis activity. The activity was seen with chloroform and methanol extracts of *P. stellatum* and *O. integrifolia* as well as with methanol and acetone extracts of *P. americana*. In addition, fractions from the most active plant, *P. stellatum* had demonstrated promising antimycobacterial activity [8].

MICs of all experimental plant extracts and solvent fractions of the most active plant extract were determined by CFU method and microplate resazurin assay method. As evidenced from Tables 2 and 3, *P. stellatum* chloroform extract was endowed with lower MIC by CFU method than by microplate resazurin assay method, although test organisms were different. It has been reported that the MIC exhibited by a compound/extract depends on the technique used for determination. MICs obtained in a liquid medium are lower than that obtained from a solid medium, as the drug has to diffuse through the matrix in the solid medium in order to exert activity [20].

MIC of *P. stellatum* chloroform extract was evaluated using CFU and Resazurin indicator method. The finding in the later method revealed that the MICs were 0.078, 0.156 and 0.156 mg/ml against AOZ8W-4, AOA8W-4 and SO38SW-4 MDR-TB clinical isolates, respectively. However, the extract had lower MIC (0.039 mg/ml) against the standard strain. The difference in MICs might be ascribed to the difference in susceptibility of the standard strain and MDR-TB clinical isolates towards the extract. Furthermore, this apparent difference might be attributed to more favorable growth conditions provided by agar culture medium to extract-treated bacilli in colony count method or the drug susceptible standard strain and the drug resistant strain might have different growth conditions. Another cause for the different MICs of *P. stellatum* chloroform extract could be the slight difference in sensitivities of colony count method and resazurin indicator method as reported by Taneja and Tyagi [26]. According to this study, Resazurin indicator assay was noted to be superior to the colony count assay in that it distinguished between metabolically active dormant bacteria and non-viable organisms, unlike the colony count assay that could not differentiate between these two populations.

In this study, *P. stellatum* chloroform extract showed promising activity against *M. tuberculosis* H37Rv and AOZ8W-4, with complete inhibition at 0.039 mg/ml and 0.078 mg/ml, respectively. These values are within the range stated by the Clinical and Laboratory Standards Institute [27]. In addition, the MDR-TB clinical isolates were resistant to isoniazid, while this plant was active against the clinical isolates of MDR-TB, possibly suggesting that the plant could have an obvious advantage over the standard drug. Moreover, the activity displayed by this extract makes the plant to be a promising plant according to Tosun et al. [28], which states that compounds with an MIC of less than 10 μg/ml, and ideally less than 2 μg/ml could have a potential for further investigation.

There is sparse data in the literature about the antimycobacterial activity of *P. stellatum*, making comparison a bit difficult. A study reported that 80% ethanol leaf extract of the plant had shown activity (MIC = 250 mg/ml) on *M. tuberculosis* H37Rv [9]. This value is much higher than the value obtained in this study, possibly indicating that non-polar constituents might be more responsible for the observed antitubercular activity. Efforts made to compare activity of the chloroform extract of *P. stellatum* with other plants showed either better or comparable activity. Activity was better compared to stem bark extract of *Anogeissus leiocarpus* [13] and comparable to that of the chloroform leaf extract of *Byrsonima fagifolia* [29].

Although 80% methanol extract of *P. stellatum* (MIC = 0.312 mg/ml), *P. americana* (MIC = 2.5 mg/ml) and *O. integrifolia* (MIC = 0.312 mg/ml) as well as chloroform extract of *O. integrifolia* (MIC = 0.312 mg/ml) and acetone extract of *P. americana* (MIC = 1.25 mg/ml) had significantly higher activity than vehicle-treated controls, they failed to exhibit promising activity according to Tosun [30] or Sánchez [31]. The MICs in this finding are lower/equal than a study done on methanol extract of *Pelargonium sidoides* (MIC = 5000 μg/ml) as well as methanol extract of *Capparis brassi*, *Entada africana* and *Combretum* species (MIC = 1250 μg/ml each) against *M. tuberculosis* H37Rv [12]. However, the MICs were higher than that of chloroform extract of *Byrsonima fagifolia* (MIC = 62.5 μg/ml) [29]. This difference might be imputed to the fact that different plant species may contain different active constituents at varying amount and/or different in vitro methods were used.

Previous studies reported several plants with promising anti-tubercular activity [13, 29–33]. Studies had mostly reported activity in the plant families of Asteraceae, Lamiaceae, Fabaceae and Apiaceae, among others [34]. It is noticeable from the present study that plants exhibiting activity belong to Fabaceae, Lamiaceae and Lauraceae families, which is in agreement with the plant families previously reported [34].

Antimycobacterial activity evaluation revealed that *P. stellatum* chloroform root extract to have a better activity and further fractionation was pursued and activity of the fractions was evaluated. Accordingly, the ethyl acetate fraction was found to be the most active fraction, based on MIC values (0.195 μg/ml), which indicated that antimycobacterial constituents were contained in this fraction. Further work is underway to isolate and characterize active principles from this fraction.

## Conclusion

The results of this study showed that *P. stellatum, O. integrifolia* and *P. americana* are endowed with a significant antimycobacterial activity. However, it seems that the potential to develop new compounds against both drug susceptible as well as MDR-TB rests with *P. stellatum*. The ethyl acetate fraction of this plant was the most active solvent fraction and further study is underway with this fraction. Although the present study provided evidence for the traditional use of some of the plants for treatment of TB, the evidence did not support the use of *V. amygdalina* and *C. edulis* for the same.

## Abbreviations

ADC: Albumin dextrose complex
AIDS: Acquired immune deficiency syndrome
ANOVA: Analysis of variance
CFU: Colony forming unit
DMSO: Dimethyl sulphoxide
HIV: Human immunodeficiency virus
MDR-TB: Multidrug resistant tuberculosis
MIC: Minimum inhibitory concentration
TB: Tuberculosis
WHO: World Health Organization
XDR-TB: Extremely drug resistant tuberculosis
